# Development of a framework to facilitate a data assembly plan for multi-regional research

**DOI:** 10.23889/ijpds.v9i5.2501

**Published:** 2024-09-18

**Authors:** Ali Anis, Carrie-Anne Whyte, Carmen La, Jean-François Ethier, Mark McGilchrist

**Affiliations:** 1 Canadian Institute for Health Information; 2 Université de Sherbrooke; 3 University of Dundee

A network of organizations works together to facilitate multi-regional research across Canada. This network is streamlining the traditionally burdensome data access process, a major part of which is a project’s data assembly plan (DAP). A framework is proposed for the development of a DAP usable by researchers and multiple data centres across Canada.

The network of 13 provincial/territorial and pan-Canadian data centres collaborated to understand variations in data request processes and local requirements. During this collaboration, partners used an iterative approach to review local forms, processes, and undertake consultations with the research community, and to identify critical components of the DAP.

In April 2022, the network launched the centrally provisioned, standardized DAP form, which to date has been deployed by approximately five projects. Users have found the DAP’s unique aggregation and documentation capabilities particularly helpful. Overall, the preliminary feedback from researchers has been positive. The DAP has allowed aggregation of specific details about a project’s data requirements: cohort definition(s), data extraction(s) and analytical plans, in a single unified form.

The DAP is an important component in streamlining the process for requesting data from multiple provinces/territories, organizations, and data sources. The DAP has ensured consistency across the network’s data centres that are providing data, supporting data linkage, and helping safeguard the quality of analytical results. Further process improvements are anticipated to address user experience feedback and to promote quality research.

